# A Methanol Extract of *Scabiosa atropurpurea* Enhances Doxorubicin Cytotoxicity against Resistant Colorectal Cancer Cells In Vitro

**DOI:** 10.3390/molecules25225265

**Published:** 2020-11-12

**Authors:** Imene Ben Toumia, Mansour Sobeh, Marco Ponassi, Barbara Banelli, Anas Dameriha, Michael Wink, Leila Chekir Ghedira, Camillo Rosano

**Affiliations:** 1 Unit of Bioactive Natural Substances and Biotechnology UR17ES47, Faculty of Dental Medicine of Monastir, University of Monastir, Avicenne Street, 5000 Monastir, Tunisia; leila.chekir@laposte.net; 2 Institute of Pharmacy and Molecular Biotechnology, Heidelberg University, Im Neuenheimer Feld 364, 69120 Heidelberg, Germany; mansour.sobeh@um6p.ma (M.S.); anas-dameriha@hotmail.com (A.D.); wink@uni-heidelberg.de (M.W.); 3 IRCCS Policlinico San Martino, Largo R. Benzi 10, I-16132 Genova, Italy; ponassi@gmail.com (M.P.); banellib.epigenetics@gmail.com (B.B.); 4 AgroBioSciences Research, Mohammed VI Polytechnic University, Lot 660–Hay MoulayRachid, 43150 Ben-Guerir, Morocco

**Keywords:** *Scabiosa atropurpurea*, doxorubicin, Caco-2, multidrug resistance

## Abstract

Colorectal cancer is a malignancy with a high incidence. Currently, the drugs used in chemotherapy are often accompanied by strong side effects. Natural secondary metabolites can interfere with chemotherapeutic drugs and intensify their cytotoxic effects. This study aimed to profile the secondary metabolites from the methanol extract of *Scabiosa atropurpurea* and investigate their in vitro activities, alone or in combination with the chemotherapeutic agent doxorubicin. MTT assay was used to determine the cytotoxic activities. Annexin-V/PI double-staining analysis was employed to evaluate the apoptotic concentration. Multicaspase assay, quantitative reverse transcription real-time PCR (RT-qPCR), and ABC transporter activities were also performed. LC-MS analysis revealed 31 compounds including phenolic acids, flavonoids, and saponins. *S. atropurpurea* extract intensified doxorubicin anti-proliferative effects against resistant tumor cells and enhanced the cytotoxic effects towards Caco-2 cells after 48 h. The mRNA expression levels of Bax, caspase-3, and p21 were increased significantly whereas Bcl-2 expression level was decreased. Furthermore, the methanol extract reversed P-glycoprotein or multidrug resistance-associated protein in Caco-2 cells. In conclusion, *S. atropurpurea* improved chemosensitivity and modulated multidrug resistance in Caco-2 cells which makes it a good candidate for further research in order to develop a new potential cancer treatment.

## 1. Introduction

Cancer is a world threat with nearly 9 million cancer-related deaths globally. Colorectal cancer (CRC) is diagnosed as the third most common cancer worldwide and the fourth leading cause of death related to malignancies. Globally, CRC is predicted to increase to 60% by 2030 [1]. This malignant disease is multifactorial and affected by several risk factors such as genetic, environmental factors, and lifestyle [2]. Chemotherapy is the most frequently applied therapy that demonstrated deleterious effects including 0.2–8.7% of chronic heart failure and 4–36% of cardiomyopathy among the patients [3]. During chemotherapy, drug resistance can be induced. Accordingly, higher doses are needed to achieve similar efficacy which leads to stronger side effects [4]. Drug combinations can be used as a way to synergistically potentiate the chemotherapeutic efficacy [5]. Generally, combining multiple drugs allows for the use of lower doses of conventional chemotherapeutical drugs, which may lead to the same or even higher efficacy due to synergism and lower resistance [5,6]. 

Plants and their diverse secondary metabolites, among them polyphenols, are an interesting source for the study and development of drugs to treat cancer [7]. The honeysuckle family, Caprifoliaceae, contains 42 genera, among them the genus *Scabiosa* [8]. *Scabiosa atropurpurea* has been demonstrated as a valuable source of pharmaceutical and health-promoting properties because of its antioxidant, hepatoprotective, and hypoglycemic activities. Chlorogenic acid methyl ester, luteolin, and luteolin 7-*O*-glucoside have been isolated from the total ethanol extract of aerial parts [9], whereby luteolin and chlorogenic acid were reported to show anticancer activity [10,11]. 

Despite the existence of several and valuable biological data about *S. atropurpurea* properties, the chemical constituents of its methanol extract remain uncharacterized. Furthermore, the full anticancer potential of the extract has not yet been exhaustively investigated. In the present study, we profiled the phytoconstituents of the methanol leaf extract utilizing LC-MS/MS. We determined its anticancer activities alone and as doxorubicin adjuvant in vitro against Caco-2 cells, resistant colorectal cancer cells. Additionally, the ability of the extract to modulate multidrug resistance-associated protein 1 (MRP1) and P-glycoprotein (P-gp, MDR1, or ABCB1) was explored.

## 2. Results

### 2.1. LC-MS Analysis of Scabiosa atropurpurea Leaves

**Figure 1:**
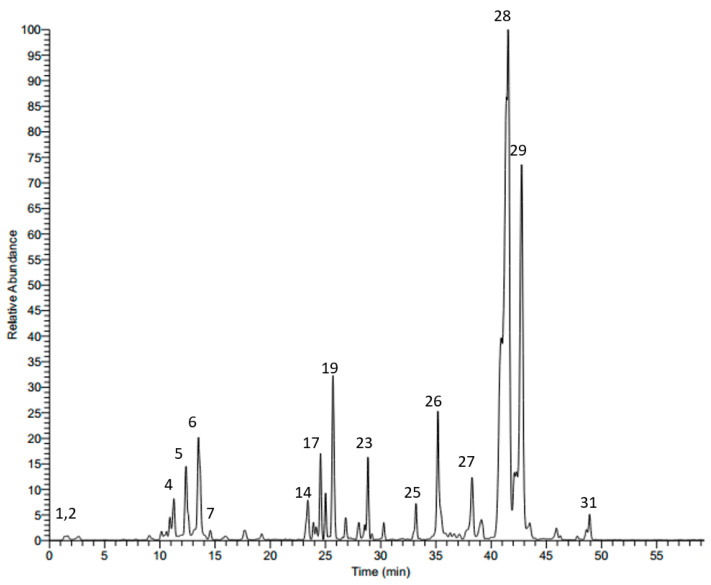
LC-MS profile of the methanol extract from *S. atropurpurea* leaves.

HPLC-MS/MS in the negative mode (ESI (−)) was used to characterize the secondary metabolites of the methanol leaf extract from *S. atropurpurea*. We tentatively identified 31 compounds based on their molecular weights and fragmentation pattern according to the literature, in-house library, and authentic references. The compounds belong to several classes: phenolic acids, flavonoids, and saponins. The LC-MS profile of the leaf extract is shown in Figure 1 and the chemical composition is presented in Table 1.

Several compounds showed a molecular ion peak at [M − H]^−^ *m*/*z* 353 and a daughter fragment at *m*/*z* 191; they were identified as chlorogenic acids, their retention times and fragmentation pattern are documented in Table 1. Furthermore, two peaks showed a molecular ion peak at [M − H]^−^ *m*/*z* 515, and a main daughter ion at *m*/*z* 353; they were identified as 3,4-dicaffeoylquinic acid (**14**) and 3,5-dicaffeoylquinic acid (**17**), respectively, and their retention times are reported in Table 1. Additionally, 3-*O*-caffeoylquinic acid methyl ester was detected at a retention time 17.62 min with a [M − H]^−^ 367, and a main precursor at *m*/*z* 191, as previously described from the same plant species from the Egyptian flora [9].

Quinic acid with an [M − H]^−^ *m*/*z* 191, protocatechuic acid 3-glucoside with an [M − H]^−^ *m*/*z* at 315 and a main fragment at *m*/*z* 153 [M − H − 162], *p*-coumaric acid 3-glucoside with [M – H]^−^ *m*/*z* at 325 and a precursor at *m*/*z* 163 [M − H − 162, glucose moiety] were also detected and identified as previously described [12].

**Figure 2:**
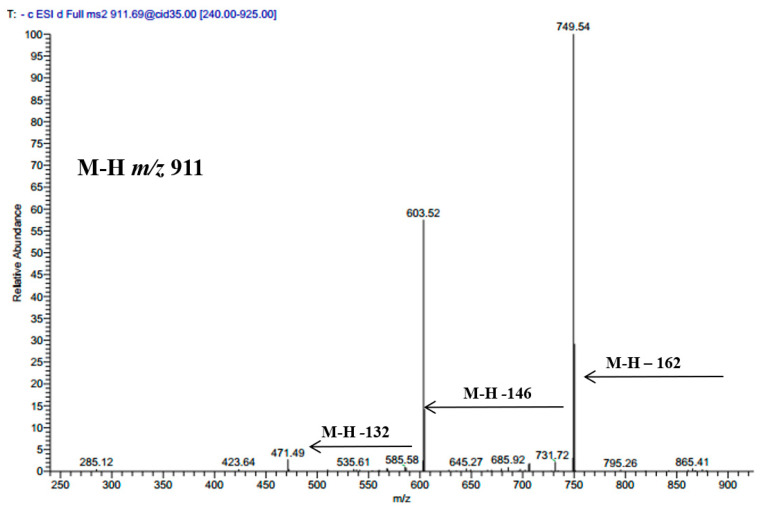
MS/MS spectrum of deprotonated compound **27** at *m*/*z* 911.

Luteolin 7-*O*-β-d-glucoside, luteolin 3’-glucoside, luteolin, and chlorogenic acid were isolated from the plant in the current study and were identified based on their LC-MS and NMR data (data are not shown). Three saponins were also annotated based on their molecular weights and mass fragmentation. A compound showed an [M − H]^−^ *m*/*z* at 911 and several fragments at 794 [M – H − 162], 603 [M − H − 162 − 146], and 471 [M − H − 162 − 146 − 132]; it was tentatively assigned to maslinic acid-pentosyl-rhamnosyl-glucoside; its spectrum is shown in Figure 2. Another compound showed a molecular ion peak at 1351 and fragments ions at 1027 [M − H − 324], 865 [M − H − 324 − 162], 733 [M − H − 324 − 162 − 146], 587 [M − H − 324 − 162 − 146 − 132]; it was tentatively identified as oleanolic acid-pentosyl-rhamnosyl-pentosyl-glucosyl-di-glucoside. Oleanolic acid-pentosyl-rhamnosyl-glucosyl-glucosyl-di-glucoside was also characterized based on its M − H *m*/*z* 1381 and subsequent loss of di-glucoside at *m*/*z* 1057 [M − H − 324], *m*/*z* 895 [M − H − 324 − 162], *m*/*z* 733 [M − H − 324 − 162 − 162], *m*/*z* 587 [M − H − 324 − 162 − 162 − 132]. Two precursors showed M − H *m*/*z* at 537 and fragment ion at *m*/*z* 375; these were assigned to loganic acid glucoside, their retention times are documented in Table 1.

### 2.2. S. atropurpurea Enhances Dox Cytotoxicity against Caco-2 MDR Colorectal Cancer

**Figure 3:**
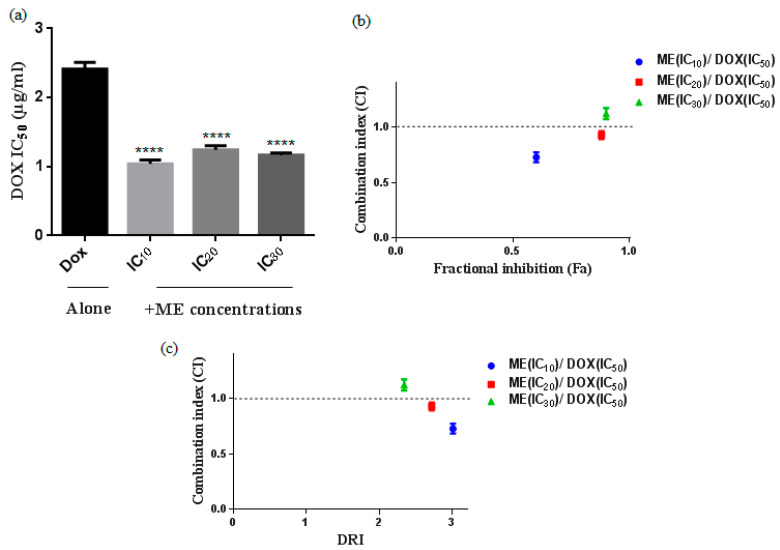
Cytotoxic effect of doxorubicin (Dox) alone or in combination with ME (IC_10_, IC_20,_ and IC_30_) against Caco-2 cells for 48 h. (**a**) IC_50_ was analyzed by MTT assay **** *p* < 0.0001 compared to the untreated DMSO control by one-way ANOVA. (**b**) The combination index (CI) and (DRI) plots were constructed according to the data obtained from the CompuSyn reporting ME (IC_10_, IC_20,_ and IC_30_) and Dox combinations. CI values indicate additive (CI = 1), synergistic (CI < 1), and antagonistic (CI > 1) effects. (**c**) Fractional response (Fa). Data represent the mean ± standard deviation of the mean of 3 independent experiments.

We evaluated the cytotoxicity of doxorubicin (Dox) in the heterogeneous human epithelial colorectal adenocarcinoma Caco-2 cell line 48 h after drug treatment using the viability assay MTT. Then, we tested the combination of Dox with fixed concentrations of the methanol extract of *S. atropurpurea* (ME) at the concentrations corresponding to the IC_10_, IC_20_ and IC_30_ doses. We observed stronger cytotoxicity with a significant decrease in IC_50_ when using a Dox-ME combination as compared to Dox alone (Figure 3a). The IC_50_ value when Caco-2 cells were treated with Dox alone is 2.41 µg/mL. The addition of ME (IC_10_) decreased the IC_50_ value to 1.04 µg/mL by 2.31-fold. The combinations of Dox with either IC_20_ or IC_30_ of ME gave almost the same effect as with IC_10_ of ME (Figure 3a).

Synergistic combinations were obtained with Dox and IC_10_ or IC_20_ or IC_30_ of ME. The combination with IC_10_ of ME and doxorubicin showed the strongest synergic effect and greater fractional inhibition (Fa) values (Figure 3b) and higher reduction index (DRI) (Figure 3c) suggesting that ME (IC_10_) is an effective dose that increases the therapeutic effect of doxorubicin against Caco-2 cells. We calculated the mean of the combination index (CI) from data points according to the Chou-Talalay method [13]. Drug combinations were chosen as the Fa > 0.5, indicating more than 50% growth inhibition, which is relevant for anticancer agents to therapy; all combinations with Fa < 0.5 were excluded.

### 2.3. Apoptotic Effect of ME and Dox against Caco-2 cells

**Figure 4:**
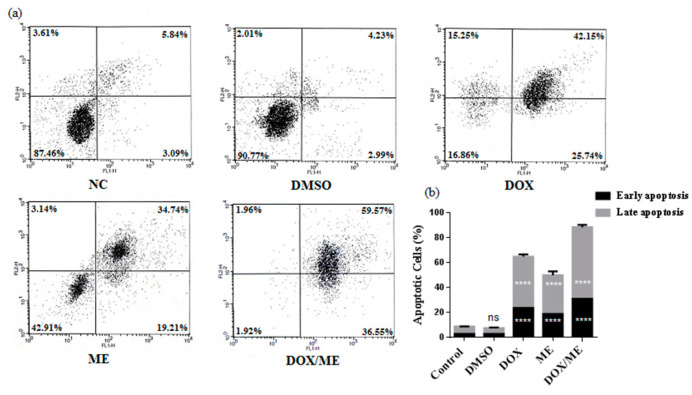
Apoptosis induction in Caco-2 cells. FACS histograms (**a**) and quantitative analysis of early and late apoptosis (**b**). Cell populations were stained with Annexin V/PI (the lower left quadrant demonstrates viable cells, upper left quadrant shows early apoptotic, the upper right quadrant represents late apoptotic and the lower right quadrant shows necrotic cells populations). Cells were treated for 48 h with Dox (IC_50_) or ME (IC_50_) or with the combination of ME (IC_10_) and Dox (IC_50_). Data represent the mean ± standard deviation of the mean of 3 independent experiments; ns (not significant), **** *p* < 0.0001 compared to the untreated DMSO control by one-way ANOVA.

We analyzed the apoptotic potential in the Caco-2 cell line with Annexin V/PI assay of ME (IC_50_) or Dox (IC_50_) or ME (IC_10_) combined Dox (IC_50_). The results showed a stronger apoptotic effect compared to Dox alone, which confirm data predicted by the combination assay (Figure 4).

### 2.4. Multicaspase Assay

**Figure 5:**
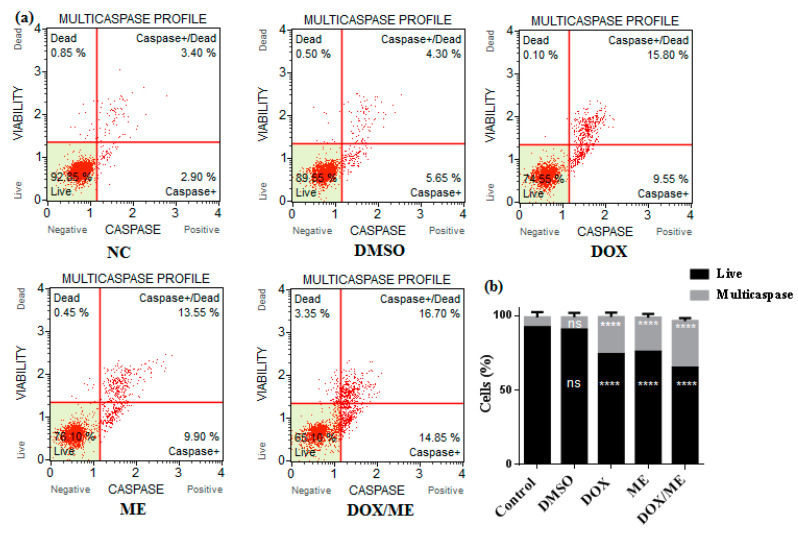
Activation assays of Multicaspase enzyme. (**a**) The plots illustrate the effect induced by the treatment with Dox (IC_50_) or ME (IC_50_) or ME (IC_10_) combined Dox (IC_50_) in the Caco-2 cell line after 24h. Each plot is a representative determination figure of the three replicates. (**b**) The bar charts depict the percentage of live cells and multicaspase enzyme activation. Data represent the mean ± standard deviation of the mean of 3 independent experiments; ns (not significant), **** *p* < 0.0001 compared to the untreated DMSO control by one-way ANOVA.

We used a Muse™ multicaspase (Millipore Merck, Vimodrone MI, Italy) assay kit to get an insight into the apoptosis mechanism of action. Initiator and executioner caspase activation (caspase-1, 3, 4, 5, 6, 7, 8, and 9) was evaluated. Data indicated that ME (IC_50_) or Dox (IC_50_) or ME (IC_10_) combined Dox (IC_50_) were able to increase activated caspases in Caco-2 cells after 24 h (Figure 5). Dox or ME or combined treatments on Caco-2 cells significantly increased the percentage of caspase-activated cells, whereby the percentages of cells were 2.53-, 2.35- and 3.17-fold higher than the corresponding vehicle DMSO control, respectively.

### 2.5. Quantitative Real Time PCR (qPCR) for Bax, Bcl-2, Casp-3 and p21 Expression in Caco-2 Cells

**Figure 6:**
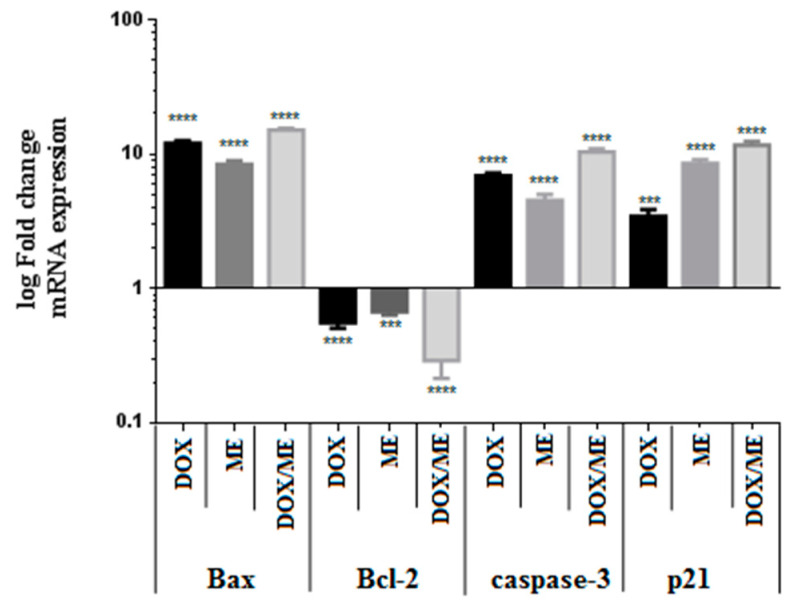
Histograms of the fold change in mRNA expression of Bax, Bcl-2, caspase-3, and p21 in Caco-2 cells. The mRNA levels of the apoptosis-related genes were determined corresponding to the housekeeping gene β-actin. The results were obtained from three independent experiments. *** *p* < 0.001, **** *p* < 0.0001.

We confirmed, based on the previous results, that ME in combination with Dox increased cytotoxicity against Caco-2 cells by inducing apoptosis. To characterize the corresponding signaling pathways, we studied the ratios of pro-apoptotic and antiapoptotic gene changes in mRNA expression levels of Bax and Bcl-2 families. As shown in Figure 6, ME combined with Dox resulted in a stronger down-regulation of the expression of the antiapoptotic gene Bcl-2 in Caco-2 cells, with a significant decrease after 48 h treatment. mRNA expression levels were calculated based on the calibration curve for the expression level of β-actin. Furthermore, the treatment with ME or Dox alone or their combination demonstrated an increase in proapoptotic gene Bax expression in Caco-2 cells. The analysis showed that the expression levels of caspase-3 (Casp-3) increased by ten-fold in cells treated with the combination drugs in a significant manner compared with untreated control (from 1 to 10.31 ± 0.64-fold change) (Figure 6). Besides, it appears that the mRNA level of p21 was upregulated after treatment with ME alone and when combined with Dox (Figure 6).

### 2.6. ME Inhibited ABC Transporter Activity

**Figure 7:**
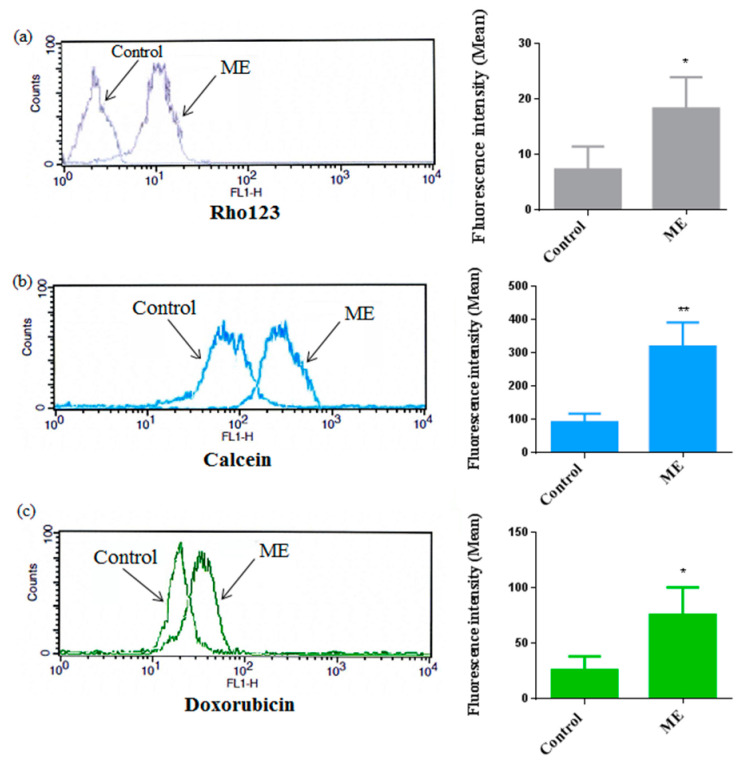
Cellular uptake of Rho123 and calcein by Caco-2 cells after treatment with ME (IC_50_) for 2 h; data are illustrated as a representative measurement of the accumulation (on the right column of the figure), and the histograms of the average of fluorescence intensity (left column) of Rho123 (**a**), calcein (**b**) and doxorubicin (**c**); cells treated with 0.1% DMSO were used as a negative control. The values are reported as an average of measurements mean ± standard deviation of approximately 10,000 cells in each triplicate tested sample. * *p* < 0.05; ** *p* < 0.01 compared to the negative control.

To gain an insight into the possible modulation of ABC-transporter activity in Caco-2 cells after ME treatment, we used both substrates for MDR1 and MRP1 for multidrug resistance inhibition assay. Rhodamine123 (Rho123), calcein-AM and doxorubicin are accumulated intracellularly when the ABC transporters are inhibited. We evaluated the effect of ME (IC_50_) on ABC transporter activity in Caco-2 cells as demonstrated in Figure 7. We found that after ME treatment the accumulation of Rho123, calcein-AM and doxorubicin in Caco-2 cells was increased significantly; the results of flow cytometry presented above showed that ME affects P-gp and MRP1 activities compared to DMSO control (<0.1%). The fluorescence intensities of Rho 123, doxorubicin and calcein-AM were shifted to the right after ME treatment.

## 3. Discussion

Medicinal plants have the potential to be used as adjuvants to chemotherapy thanks to their proven efficacy and protective action against the induced chemo-damages [14,15]. Previous studies have demonstrated that the genus, *Scabiosa*, exhibited differential cytotoxic effects on cancer cell lines [16]. In the current study, the chemical composition of the extract revealed 31 secondary metabolites including phenolic acids, flavonoids, and saponins. The extract exhibited anticancer activities alone and in combination with the cytotoxic drug, doxorubicin.

In order to evaluate this anti-cancer mechanism of action, we tested ME in resistant colon cancer cell lines. ME (IC_10_) combined with Dox resulted in lower IC_50_ in comparison to Dox alone. These results suggest that ME may be a potential adjuvant and could be involved in cell death. We demonstrated that ME induced early and total apoptosis in Caco-2 cells and also showed that a non-toxic concentration of ME induced the highest level of programmed cell death when combined with Dox. These results confirmed that Caco-2 cells are more sensitive to the combination of conventional chemo-drugs with phytocompounds [17].

Cell apoptosis represents an energy-dependent process of programmed cellular death. DNA fragmentation, cell shrinkage and chromatin condensation are associated with this mechanism [18,19]. Apoptosis is mediated via two main pathways by intracellular and extracellular signals which include the two pathways of mitochondrial apoptosis and death receptor-mediated [20,21]. We determined the apoptosis pathway of ME alone or as adjuvant by total caspases activities. ME increased caspase activities by 50% and 30%, respectively. In a previous study, caspase dependence has been observed for luteolin, a major secondary metabolite isolated from the plant [22].

In the same way, the induction of apoptosis by natural compounds may have been orchestrated by mediating proteins such as Bax and Bcl-2 and caspase-3, as well as by the processes of cell cycle arrest [23]. We have also shown that the cell apoptosis induction is occurring in response to various stress conditions and it includes the regulation of the p21 gene, which is one of the major tumor suppressor p53 targets, a dependent p21 nuclear translocation that could promote cell survival and inhibits CDK2 preventing cell cycle progression [24]. In this study, we demonstrated a decrease in Bcl2 expression and an increase in Bax, caspase-3 and p21. These results could be attributed to single or combined activities of various active polyphenols identified in the methanol extract of *S. atropurpurea*, such as chlorogenic acid, which improved the mediated cell death when combined with Dox in solid Ehrlich carcinoma (SEC) model in mice. Chlorogenic acid alone or with Dox regulated the expressions of TRAIL/TRAILR2, caspase-3, FasL/Fas and Bcl-2 [25].

Additionally, the antitumor agents of chemotherapy are compromised by several mechanisms, such as ATP-binding cassette (ABC) transporters, which efflux chemotherapeutical drugs out of cells. The hydrophobic drug export used the energy of ATP binding across the cell membrane and limited the concentration of the intracellular drug. As previously described, P-gp and MRP were modulated by plant extract. Particularly, Dox or combinations of plant extracts examined the intracellular retention of the ABC transporter substrates then explored the changes in the expression genes of ABCB1 and ABCC1 [7]. The cooperation of different compounds was sufficient to improve the activities and to lead to a better impact than the separate potentials, by resulting in a synergistic effect. This strategy has evolved by nature to obtain better efficacy at lower energetic costs [26]. In this regard, the potent effects of ME could be correlated to the synergistic effect of some of the identified compounds.

## 4. Materials and Methods

### 4.1. Plant Material

*S. atropurourea* L. subsp. *maritima* (L.) Arc leaves were collected from Sidi Alouane, Mahdia, Tunisia during the spring season in May 2018. The plant identity was kindly confirmed by Prof. Fethia Skhiri (ISBM, Monastir, Tunisia). A voucher specimen (CP# 1783) is kept at the Department of Pharmacognosy, Faculty of Pharmacy, Monastir University.

### 4.2. Extraction

The shade dried leaves of *S. atropurpurea* (200 g) were ground into fine powder by an electric mill and extracted twice with methanol (2 × 1 L) with occasional shaking at room temperature for three days. The combined extracts were filtered and concentrated at 40 °C to yield 56 g of the crude extract (yield: 28% *w*/*w*). The resulting extract was defatted with hexane, frozen, and lyophilized yielding a fine powder (31 g) then stored at −20 °C for chemical and biological investigations. In each of our assays, we dissolved ME and Dox in DMSO (100%) then the solutions were diluted in a complete medium. The treatments were achieved at final volume of DMSO (0.1%).

### 4.3. Phytochemical Analysis

LC-MS/MS analysis was performed on a Thermo Finnigan LCQ Advantage ion trap mass spectrometer with an ESI source coupled to a Thermo Scientific Accela HPLC system (MS pump plus, autosampler, and PDA detector plus) with an EC 150/2 Nucleodur 100-3 C18ec column (Macherey-Nagel). A gradient of water and acetonitrile (ACN) was applied from 5% to 30% ACN in 60 min. The flow rate was 0.3 mL/min. The MS was operated in the negative mode with a capillary voltage of 10 V, source temperature of 240 °C, and high purity nitrogen as a sheath and auxiliary gas at a flow rate of 70 and 10 (arbitrary units), respectively. Data acquisitions were executed by Xcalibur^TM^ 2.0.7 software (Thermo Scientific). The ions were detected in a mass range of 50–2000 *m*/*z*.

### 4.4. MTT Assay

Caco-2 cells were maintained in DMEM supplemented with heat-inactivated fetal bovine (FBS) 10% (*v*/*v*), penicillin (100 U/mL), streptomycin (100 µg/mL), l-glutamine (2 mM), non-essential amino acids (NEAA) 1% (*v*/*v*) and sodium pyruvate (1 mM). The cytotoxic effects were performed using MTT assay. ME and Dox were tested at different concentrations ranging from 0 to 500 μg/mL and 0 to 50 μg /mL, respectively. Briefly, Caco-2 cells were plated at a density of 5 × 10^3^ cells/well were cultured in 96-well plates for 48 h. After incubation, MTT (0.5 mg/mL) was added then incubated for 4 h. The absorbance was determined at 570 nm using Eliza Plate Reader. IC_50_ values were calculated with GraphPad Prism 6.01 software (GraphPad Software Inc., La Jolla, CA, USA).

### 4.5. Drug Combination Assays

Drug combinations against Caco-2 cells were evaluated using three non-toxic concentrations (IC_10_, IC_20,_ and IC_30_) of ME combined with Dox (IC_50_). Cells were incubated briefly into 96-well plates, MTT assay was carried as mentioned above. We assessed the effect of drug interactions (additive, synergistic or antagonist) using the method of CI, DRI, and Fa as described by the Chou method [13,27].

### 4.6. Annexin-V/PI Double-Staining Analysis of Apoptotic Cells

Caco-2 cells (1 × 10^6^ cells/mL) were treated for 48 h with ME (IC_10_) and Dox (IC_50_). Annexin-V/PI (25 ng/mL) (Roche Diagnostics GmbH, Mannheim, Germany) was then added. Next, propidium iodide (PI) (50 ng/mL) was added to the mixture. We evaluated cell status with a FAC Scan flow cytometer (Becton Dickinson, Heidelberg, Germany) and calculated data using Cell QuestTM Pro software (Becton Dickinson).

### 4.7. Multicaspase Assay

Caco-2 cells (1 × 10^6^ cells/well/ 2 mL culture media) were seeded at 37 °C in a 6-well plate, when cells reached 70% of confluence, we treated them with Dox (IC_50_) or ME (IC_50_) or ME (IC_10_) combined Dox (IC_50_) for 24 h. A Muse™ multicaspase assay kit (Millipore Merck, Vimodrone MI, Italy) was used for the detection of multiple caspase activation (Caspase-1, 3, 4, 5, 6, 7, 8 and 9), following the manufacturer’s instruction. Then, the percentage of cells with multicaspase activity was examined using a Muse™ cell analyzer (Millipore Merck, Vimodrone MI, Italy).

### 4.8. Quantitative Reverse Transcription Real-Time PCR (RT-qPCR)

Caco-2 cells were treated with ME and Dox at different concentrations to examine the mRNA expressions. RNA extraction was performed with RNeasy Midi Kit (Qiagen, Hilden, Germany) and RT-qPCR was performed as recommended by Lightcycler 96 manufacturer protocol (Roche, Germany) with a PCR master mix with SYBR Green I fluorescence (qPCRBIO SyGreen Mix Lo-Rox, Nippon Genetics, Germany) as described by Su et al. [28], and by using the following gene-specific primers obtained from Eurofins (Germany): Bax sense (5′-GTCGCCCTTTTCTACTTTGC-3′), Bax antisense (5′-GGAGGAAGTCCAATGTCCAG-3′), Bcl-2 sense (5′-GGATTGTGGCCTTCTTTGAG-3′), Bcl-2 antisense (5′-GCCGGTTCAGGTACTCAGTC-3′); p21 sense (5′-GACTCTCAGGGTCGAAAACG-3′) and p21 antisense (5′-GGATTAGGGCTTCCTCTTGG-3′), Caspase 3 sense (5′-GAGCTTGGAACGCGAAGAAA-3′), Caspase 3 antisense (5′-TAACCGGGTGCGGTAGAGTA-3′), β-actin sense (5′-CGCCCCAGGCACCAGGGC-3′), β-actin antisense (5′-GCTGGGGTGTTGAAGGT-3′). We normalized the relative expression levels to the transcription level of the housekeeping β-actin gene. Finally, we used the Software LightCycler 3 Version 3.5.28 (Roche Applied Science; Mannheim, Germany) for data analysis. Data were represented as fold change with respect to untreated cells (0.1% DMSO) and fold change represented in log 10 scale.

### 4.9. ABC Transporter Activity

The colorectal cancer cell line, Caco-2 cells, functionally expresses P-gp and MRP1. ABC transporter activities were examined using rhodamine 123 (Rho123), calcein-AM (CAM) and Dox using flow cytometry as previously described [29]. Briefly, Caco-2 cells were seeded at 1 × 10^5^ cells/well in 6-well plates. Then, Caco-2 cells were incubated with 2 µM of CAM or 10 µg/mL Rho123 or 2.41 µg/mL Dox for 2 h, followed by 2 h with the test sample ME (IC_50_). The fluorescence intensity of the treated cells with ME was compared to the untreated cells.

### 4.10. Statistical Analysis

We performed all assays three times as stated in the procedure. All data are represented as mean ± standard deviation. Graphs were drawn using GraphPad Prism 6.01 software (GraphPad Software Inc., La Jolla, CA, USA). One-way analysis of variance was used to evaluate sets of data differences. A *p*-value of less than 0.05 was considered significant.

## 5. Conclusions

LC-MS/MS profiling of the methanol extract of *S. atropurpurea* revealed three basic classes of compound phenolic acids, flavonoids and saponins among 31 secondary metabolites. The extract exhibited promising anticancer activities in vitro, both alone and in combination with doxorubicin. Cell proliferation was significantly suppressed. The apoptotic cell rate increased when we combined the extract with doxorubicin. In conclusion, *S. atropurpurea* has the potential to be a multidrug resistance modulator candidate. It may be an attractive alternative to chemosensitize the doxorubicin effect by enhancing its efficacy in colorectal cancer cells. The pharmacological features of *S. atropurpurea* as adjuvant needs to be investigated in a more detailed study. It would be interesting to exploit the synergistic effect of the compounds from *S. atropurpurea* to avoid possible adverse side effects and to achieve better outcomes of anticancer treatment.

## Figures and Tables

**Table 1:** Polyphenolics in a methanol extract of *S. atropurpurea* leaves.

No.	Rt	M − H	MS/MS	Tentatively Identified Compound
**1**	1.43	191	127, 173	Quinic acid ^a^
**2**	7.71	315	153	Protocatechuic acid 3-glucoside ^a^
**3**	10.35	325	119, 163	*p*-Coumaric acid 3-glucoside ^a^
**4**	11.28	353	179, 191	Neochlorogenic acid
**5**	12.46	353	179, 191	Cryptochlorogenic acid
**6**	13.53	353	179, 191	Chlorogenic acid ^a,b^
**7**	14.28	373	149, 193	Ferrulic acid derivative
**8**	15.82	625	301, 463	Quercetin diglucoside
**9**	16.07	337	119, 163, 191	*p*-Coumeroylquinic acid ^a^
**10**	17.12	609	285, 327, 447	Luteolin-7,3-diglucoside ^a^
**11**	17.52	625	179, 255, 301, 463	Quercetin 3,4’-diglucoside ^a^
**12**	17.62	367	127, 173, 191	3-*O*-Caffeoylquinic acid methyl ester
**13**	19.16	609	285, 327, 447	Luteolin-7,3’-diglucoside ^a^
**14**	22.95	515	353	3,4-Dicaffeoylquinic acid
**15**	23.35	579	285, 447	Luteolin-pentosyl-glucoside
**16**	23.89	579	285, 447	Luteolin-pentosyl-glucoside
**17**	24.43	515	179, 353	3,5-Dicaffeoylquinic acid
**18**	25.05	593	285, 447	Luteolin 7-rutinoside ^a^
**19**	25.71	447	285	Luteolin 7-*O*-*β*-d-glucoside ^b^
**20**	26.88	447	179, 285	Luteolin 3’-glucoside ^b^
**21**	27.79	593	299	Diosmetin pentosyl-glucoside
**22**	28.12	745	583	Cantleyoside
**23**	28.60	431	269	Apigenin 7-glucoside ^a^
**24**	30.28	461	299	Diosmetin-7-*O*-glucoside
**25**	33.18	489	285, 447	Luteolin acetylglucoside
**26**	35.22	285	151	Luteolin ^b^
**27**	38.48	911	471, 603, 749	Maslinic acid-pentosyl-rhamnosyl-glucoside
**28**	41.24	1351	455, 587, 733, 865, 1027	Oleanolic acid-pentosyl-rhamnosyl-pentosyl-glucosyl-di-glucoside
**29**	41.70	1381	455, 587, 733, 895, 1057	Oleanolic acid-pentosyl-rhamnosyl-glucosyl-glucosyl-di-glucoside
**30**	46.15	537	375	Loganic acid glucoside
**31**	49.11	537	375	Loganic acid galactoside

^a^ Identified based on reference compounds. ^b^ Isolated from the plant.
